# Reviewer acknowledgement 2012

**DOI:** 10.1186/1471-2334-13-50

**Published:** 2013-04-02

**Authors:** Philippa K Harris

**Affiliations:** 1 BioMed Central, 236 Gray’s Inn, Road, London, WC1X 8HB, UK

## Abstract

**Contributing reviewers:**

The editors of BMC Infectious Diseases would like to thank all our reviewers who have contributed to the journal in Volume 12 (2012).

## 

Sayed Abdelwahab

Egypt

Juraina Abd-Jamil

Malaysia

Fekadu Abebe

Norway

Pierre Abgueguen

France

Oladipo Aboderin

Nigeria

Mark Abzug

USA

Hugues Adegbidi

Benin

Ayola Akim Adegnika

Gabon

Hammami Adnene

Tunisia

Brian Agan

USA

Girdhar Agarwal

India

Ritesh Agarwal

India

Dilruba Ahmed

USA

Sitara Ajjampur

India

Halis Akalin

Turkey

Stella Akinleye

Nigeria

Norio Akuta

Japan

Mahboob Alam

USA

Abdurhman Alarfaj

Saudi Arabia

Seyed-Moayed Alavian

Iran

Jan Albert

Sweden

Werner C. Albrich

Switzerland

Samim Al-Dabbagh

Iraq

Sahal Al-Hajoj Al-Nakhli

Saudi Arabia

Majdi Al-Hasan

USA

Hammad Ali

Australia

Jean-Pierre Allain

UK

Stephen Allen

UK

Paulo Almeida

Portugal

Graca Almeida-Porada

USA

Jordi Almirall

Spain

Mark Almond

UK

Sylvie Alonso

Singapore

Noelia Alonso

Spain

Wladimir Alonso

USA

Ahmed Alsuwaidi

United Arab Emirates

Benjamin Althouse

USA

Eleonora Altman

Canada

Mathias Altmann

Germany

Sydney Alves

Brazil

Zhijie An

China

Kathryn Anastos

USA

Katie Anders

Australia

Monique Andesson

South Africa

Laurent Andreoletti

France

Juan Carlos Andreu-Ballester

Spain

Jason Andrews

USA

Nick Andrews

UK

Jon Kim Andrus

USA

Yadouleton Anges

Benin

Andrea Angheben

Italy

Martina Angi

UK

Brian Angus

UK

Filippo Ansaldi

Italy

Denise Antona

France

Guido Antonelli

Italy

Martinez Antonio

Spain

Beena Antony

India

Shampa Anupurba

India

Ludwig Apers

Belgium

Simon Apte

Australia

Victor H Aquino

Brazil

Michael Arabatzis

Greece

CarmenArdanuy

Spain

Roberto Arenas

Mexico

Sandra Arend

Netherlands

Maurizio Arico

Italy

Shams Arifeen

Bangladesh

Minetaro Arita

Japan

A Arivazhagan

India

Kaku Armah

USA

Elise Arrivé

France

Valentina Arsic Arsenijevic

Serbia

Rana Asghar

USA

Atif Asghar

Saudi Arabia

Ojan Assadian

Austria

Labrini Athanasiou

Greece

Katherine Atkins

USA

Linda Aurpibul

Thailand

Narong Auvichayapat

Thailand

Manuela Avolio

Italy

John Ayisi

Kenya

George Ayodo

Kenya

Cenk Aypak

Turkey

Aliyu Babadoko

Nigeria

Enrique Baca-Garcia

Spain

Prasith Baccam

USA

Steffen Backert

Ireland

John Baddley

USA

Parisa Badiee

Iran

Raffaele Badolato

Italy

Henry Baggett

Thailand

Guirong Bai

Japan

Michael Baier

Germany

Sarah Lou Bailey

UK

Maria Paula Bajanca-Lavado

Portugal

Francis Bajunirwe

Uganda

Fausto Baldanti

Italy

Pablo Baldi

Argentina

Colleen Bamford

South Africa

David Banach

USA

Jangu Banatvala

UK

Steffen Bank

Denmark

Sayera Banu

Bangladesh

Krisztian Banyai

Hungary

Ioannis Baraboutis

Greece

Jack Barker

UK

Peter Bartmann

Germany

Naor Bar-Zeev

Malawi

Partha Basu

India

Chris Bauch

Canada

David Baxter

UK

Nurcan Baykam

Turkey

David Bearden

USA

Christiane Bebear

France

Marissa Becker

Canada

Jerzy Behnke

UK

Richard Beigi

USA

Maria Bell

USA

Nancy Bellei

Brazil

Edward Belongia

USA

Ronen Ben-Ami

Israel

Bojana Beovic

Slovenia

Joseph Berger

USA

Jesus F Bermejo-Martin

Spain

Kristen Bernard

USA

Andrew David Beswick

UK

Philippe Beutels

Australia

Lothar Beutin

Germany

Charles Bevins

USA

Patricia Bezerra

Brazil

Achuyt Bhattarai

USA

Bianca Bianco

Brazil

Quentin Bickle

UK

Douglas Biedenbach

USA

Cedric Bien

USA

Matthew Biggerstaff

USA

Norma Binsztein

Argentina

Zeno Bisoffi

Italy

Melanie Bissessor

Australia

Idir Bitam

Algeria

Cecília Bittencourt Severo

Brazil

Chantal Bleeker-Rovers

Netherlands

Johannes Bogaards

Netherlands

Paolo Bonanni

Italy

Hector Bonilla

USA

Marc Bonten

Netherlands

Basmattee Boodram

USA

Mark Booth

UK

Martin Bootsma

Netherlands

Shubhada Bopegamage

Slovakia

Michael Borg

Malta

Steffen Borrmann

Germany

Ray Borrow

UK

Mridula Bose

India

Steven Bosinger

USA

Kathleen Botham

UK

Graham Bothamley

UK

Teun Bousema

UK

Vincent Bouteloup

France

Jolene Bowers

USA

John M. Boyce

USA

Cynthia Braga

Brazil

Lulu Bravo

Philippines

Ronan Breen

UK

Stéphane Bretagne

France

Carlos Brites

Brazil

Robin Brittain-Long

UK

Shobha Broor

India

Julia Brotherton

Australia

Matthijs Brouwer

Netherlands

Alison Brown

UK

Jeremy Brown

UK

Lillian Brown

USA

Brandon Brown

USA

Sylvia Bruisten

Netherlands

Nele Brusselaers

Sweden

James Brust

USA

Brita Bruun

Denmark

Kristina Bryant

USA

Ana Budimir

Croatia

Michael Bukrinsky

USA

Marloes Bults

Netherlands

Joaquin Burgos

Spain

Almudena Burillo

Spain

Colin Burnell

Canada

Rosemary Burnett

South Africa

Joseph Burzynski

USA

Patrick Butaye

Belgium

Dana Byrne

USA

Miguel Cabada

Peru

Hong Cai

China

Andrea Calcagno

Italy

Ludmila Camargo

Brazil

Helen Campbell

UK

Giuseppina Campisi

Italy

Hakan Camuzcuoglu

Turkey

Massimiliano Cantinotti

Italy

Bin Cao

China

Amedeo Capetti

Italy

Bruno Caramelli

Brazil

Anne Carbonne

France

Pere-Joan Cardona

Spain

Jean Carlet

France

PhilipCarling

USA

Yehuda Carmeli

Israel

Edoardo Carretto

Italy

Alfonso Carrillo-Muñoz

Spain

Cecilia Godoy Carvalhaes

Brazil

Scott Carver

USA

Antonio Cascio

Italy

Claudio Casoli

Italy

Corey Casper

USA

Elio Castagnola

Italy

Philip Castle

USA

Jose Catalan

UK

Maria Adriana Cataldo

Italy

Chiara Cattaneo

Italy

Nilgun Cerikcioglu

Turkey

Dimitri Ceroni

Switzerland

Dave Chadee

Trinidad and Tobago

Angkana Chaiprasert

Thailand

Romanee Chaiwarith

Thailand

Runu Chakravarty

India

James Chalmers

UK

Kian Sing Chan

Singapore

Douglas Chan

Singapore

Hsin-HouChang

Taiwan

Ding-Kwo Chang

Taiwan

John Changalucha

Tanzania

Nora Chapman

USA

Salome Charalambous

South Africa

Theeraphap Chareonviriyaphap

Thailand

Pierre-Emmanuel Charles

France

Caroline Chartrand

Canada

Sanjay Chaturvedi

India

Stylianos Chatzipanagiotou

Greece

Davendra Singh Chauhan

India

Vinita Chauhan

USA

Porntip Chavalitshewinkoon-Petmitr

Thailand

Luis Fernando Chaves

USA

Mark Cheetham

UK

Novel N Chegou

South Africa

Liliana Chemello

Italy

Mark I-Cheng Chen

Singapore

Xiang-Sheng Chen

China

Chi-Ju Chen

Taiwan

Xiang-Sheng Chen

China

Junhu Chen

China

Yen-Hsu Chen

Taiwan

Kow-Tong Chen

Taiwan

Yi-Ming Arthur Chen

Taiwan

Mark I-Cheng Chen

Singapore

Jason Chen

USA

Xiang-Sheng Chen

China

Kow-Tong Chen

Taiwan

Yixin Chen

China

Liang Chen

USA

Thomas Cherpes

USA

Chung Yan Cheung

Hong Kong

Roberto Chiavaroli

Italy

Shingo Chihara

USA

Violet Chihota

South Africa

Ng Lee Ching

Singapore

Sadegh Chinikar

Iran

Nan-Chang Chiu

Taiwan

Cheng-Hsun Chiu

Taiwan

Christinah Chiyaka

USA

Teena Chopra

USA

Ai-Hsiang Chou

Taiwan

Gerardo Chowell

USA

Jens Jørgen Christensen

Czech Republic

Denise Christofolini

Brazil

Erja Chryssanthou

Sweden

Huiming Chung

USA

Massimo Ciccozzi

Italy

Luka Cicin-Sain

Germany

Daniela M Cirillo

Italy

Milan Cizman

Slovenia

Thomas Clark

USA

Jesse Clark

USA

Gary Clifford

France

Eliana Coccia

Italy

Luis Coelho

Portugal

Juliana Coelho

UK

Ted Cohen

USA

Steven Cohen

USA

Robert Cohen

France

Robert Colebunders

Belgium

Beth-Ann Coller

USA

Phil Collier

UK

Anne Collignon

France

Arnaldo Lopes Colombo

Brazil

Guillermo Comach

Venezuela

Teresa Conceicao

Portugal

Anali Conesa-Botella

Belgium

Andrew Conway-Morris

UK

Ben Cooper

Thailand

Diane Cooper

South Africa

Yacoob Coovadia

South Africa

Ross Coppel

Australia

Teresa Coque

Spain

Alyssa Cornall

Australia

Oliver Cornely

Germany

Yvonne Cossart

Australia

Cristina Costa

Italy

David Courtin

Benin

Benjamin Cowling

Hong Kong

Alessandro Cozzi-Lepri

UK

Michael Craig

USA

Ross Cranston

USA

Christopher Crnich

USA

Julio Croda

Brazil

Bruce Cronstein

USA

Natasha Crowcroft

Canada

Brendan Crowley

Ireland

Maria Letícia Cruz

Brazil

Manuel Cuenca-Estrella

Spain

Maria de Lourdes Cunha

Brazil

Maria Paula Curado

France

**Douglas Curran**-**Everett**

USA

Scott Curry

USA

Christopher Czaja

USA

Simon Daefler

USA

Oktavija Dakovic Rode

Croatia

Lidia Dalfino

Italy

Axel Dalhoff

Gibraltar

David Dance

Laos

Prajnan Das

USA

Gokul Das

USA

Rajib Dasgupta

India

Robert Daum

USA

Michael David

USA

Fernanda de Araujo

USA

Hans de Beenhouwer

Belgium

Antonella De Donno

Italy

Cornelis PC de Jager

Netherlands

Birgitta de Jong

Sweden

Isabel de Kantor

Argentina

Louis de Léséleuc

Canada

Federico De Marco

Italy

Maurizio de Martino

Italy

Marie de Perio

USA

Andrés de Roux

Germany

Koen De Schrijver

Belgium

Jacobus de Waard

Venezuela

Nathan Dean

USA

Vincent DeBari

USA

Martin Dedicoat

South Africa

Shelley Deeks

Canada

Adriaan Dees

Netherlands

Julia del Amo

Spain

Elisabeth

Delarocque-Astagneau

France

Giovanni Delogu

Italy

Walter Demczuk

Canada

Hendrik Cornelis den Bakker

USA

Dereje Dengela

USA

Olivier Denis

Belgium

Ali Deniz

Turkey

Paul Denton

USA

Dominic Dery

Ghana

Diane Descamps

France

Jean-Claude Desenclos

France

David DeShazer

USA

Reinhard Dettmeyer

Germany

Nicolaas Deutz

USA

Aditi Dey

Australia

Abhay Dhand

USA

Amreeta Dhanoa

Malaysia

Ramesh Dhiman

India

Simona Di Giambenedetto

Italy

Ismael Diallo Burkina

Faso

Fredi Alexander Diaz-Quijano

Colombia

Roland Diel

Germany

Christine Dierkes

Germany

Jo-Anne Dillon

Canada

George Dimopoulos

Greece

Olgica Djurkovic-Djakovic

Serbia

Orsolya Dobay

Hungary

Rainer Doffinger

UK

Pere Domingo

Spain

Massimiliano Don

Italy

Michele Doran

Ireland

Susan Dorman

USA

Maria Dorrucci

Italy

Horng-Yunn Dou

Taiwan

David Dowdy

USA

Lorenzo Drago

Italy

Mignon du Plessis

South Africa

Zhaojun Duan

China

Jitender Dubey

USA

Shalini Duggal

India

Jean-Claude Dujardin

Belgium

Roger Dumke

Germany

Brigitte Dunais

France

Stacy Duncan

USA

Eileen Dunne

Australia

Jean Dupouy-Camet

France

Christine Durand

USA

Paolo Durando

Italy

Emanuele Durante Mangoni

Italy

Malcolm Duthie

USA

Joe Dylewski

Canada

Mark Ebell

USA

Tim Eckmanns

Germany

Zoe Edelstein

USA

Charles Edwards

Barbados

Philippe Eggimann

Switzerland

Jan Ehrchen

Germany

Stephan Ehrhardt

USA

Girum Ejigu

USA

Mona El Raziky

Egypt

Wael El-Tras

Egypt

Emmanuel Piednoir

France

Marieke Emonts

Netherlands

Bobbie Erickson

USA

Ananias Escalante

USA

Pedro Pablo España Yandiola

Spain

Luis Espinoza

USA

Susanna Esposito

Italy

Dean Eurich

Canada

Meirion Evans

UK

Massimiliano Fabbiani

Italy

Stephanie Factor

USA

Howard Faden

USA

DeLisa Fairweather

USA

Marco Falcone

Italy

Lars Falk

Sweden

Brian Fallon

USA

Rong Fang

USA

M. Carmen Fariñas

Spain

Albert Farrugia

USA

Gaetan Faubert

Canada

Henry Fechner

Germany

Kevin Fenton

USA

Peter Ferenci

Austria

Ricardo Fernandez Roblas

Spain

Jose Vicente Fernandez-Montero

Spain

Giovanni Ferrara

Italy

Matthew Ferrari

USA

Catterina Ferreccio

Chile

Katherine Fethers

Australia

Mark Fiandaca

USA

Magdalena Figlerowicz

Poland

Antonietta Filia

Italy

Ramona Finnie

USA

Gregory Firth

South Africa

Dale Fisher

Singapore

Elmira Flem

Norway

Fiona Fleming

UK

Thilo Floerkemeier

Germany

Andres Floto

UK

Emanuele Focà

Italy

António Pedro Fonseca

Portugal

Nathan Ford

South Africa

Christen Fornadel

USA

Jeffrey Foster

USA

Vincent Foulongne

France

Betsy Foxman

USA

**Anne Marie**-**France**

USA

Bruno Francois

France

Christopher Frei

USA

Manuel Fresno

Spain

Faten Frikha

Tunisia

Angelika Fruth

Germany

Sandra Fuchs

Brazil

Kohtaro Fujihashi

USA

Sebastian Funk

USA

Wieslaw Furmaga

USA

Guilherme Furtado

Brazil

Jon Furuno

USA

Giovanni Gabutti

Italy

Mônica Gadelha

Brazil

Naomi Gadsby

UK

Julia Gage

USA

Jean Bosco Gahutu

Rwanda

Angel S. Galabov

Bulgaria

Micheal Gallagher

Ireland

Laura Galli

Italy

Neel Gandhi

USA

Daniel Ganger

USA

Salisu Garba

South Africa

Jesus MGarcia Calleja

Switzerland

Dario Garcia de Viedma

Spain

Elisa Garcia-Vazquez

Spain

Fernando Garelli

Argentina

Bhavuk Garg

India

Paul Garner

UK

Carolina Garrido

USA

Gaëtan Gavazzi

France

Dongliang Ge

USA

Nicholas Geard

Australia

Suzanne Geerlings

Netherlands

Annemieke Geluk

Netherlands

**Wolfram H**.**Gerlich**

Germany

Gianluca Gessoni

Italy

Giovanni Gherardi

Italy

Souvik Ghosh

Japan

Reza Ghotaslou

Iran

Katherine Gibney

Australia

Sebastien Gibot

France

Phil Giffard

Australia

Ruth Gil-Prieto

Spain

Enrico Girardi

Italy

Massimo Giuliani

Italy

Anna Giuliano

USA

Erik Glocker

Germany

Kee Tai Goh

Singapore

David Goldberg

UK

David Goldberg

USA

Elad Goldberg

Israel

Daniel Goldenberger

Switzerland

George Golding

Canada

Beatriz Lucia Gomez Giraldo

Colombia

Camila Gonzalez

Colombia

Stevan Gonzalez

USA

Esteban Gonzalez Diaz

Mexico

Gloria Gonzalez-Aseguinolaza

Spain

Abraham Goorhuis

Netherlands

Patricia Gorak-Stolinska

UK

Rachel Gordon

USA

Adam L Gordon

UK

Rudra Prosad Goswami

India

Catarina Gouveia

Portugal

J.-Matthias Graf von der Schulenburg

Germany

Celso Granato

Brazil

Denis Grandgirard

Switzerland

Liliane Grangeot-Keros

France

Stephen Graves

Australia

RichardGray

Australia

Magdalena Grce

Croatia

Justin Green

UK

Pamela Greenwell

UK

Christopher Gregory

USA

Pierfrancesco Grima

Italy

Martin Peter Grobusch

Netherlands

Uwe Gross

UK

Julin Gu

China

Giovanni Guaraldi

Italy

Jeannette Guarner

USA

Sophie Gubbels

Denmark

Francisco Gude

Spain

Sebastian Guenther

Germany

Benoit Guery

France

Jesus Guinea

Spain

Javier Guitian

UK

Qi Guo

China

Ana Gutiérrez-Escolano

Mexico

Steven Haase

USA

Mario Habek

Croatia

Michele Hacker

USA

Lars Hagberg

Sweden

Andrew Hall

UK

Luanne Hall-Stoodley

USA

HenrikeHamer

Netherlands

Margaret Hammerschlag

USA

Klaus Hamprecht

Germany

Jennifer Han

USA

Tao Han

China

Werner Handrick

Germany

Madeleine Hanekom

South Africa

Marja-Liisa Hänninen

Finland

**Thomas** H**anslik**

France

Christian Happi

Nigeria

Stephan Harbarth

Switzerland

Richard Harding

UK

John Hargrove

South Africa

Diane Harper

USA

Julie Harris

USA

Ross Harris

UK

Ingunn Harstad

Norway

Habsah Hasan

Malaysia

Rumina Hasan

Pakistan

Zahra Hasan

Pakistan

Rodrigo Hasbun

USA

Toshio Hattori

Japan

Angelos Hatzakis

Greece

Timo Hautala

Finland

David Hawkins

UK

Tetsuya Hayashi

Japan

Na He

China

Hongxuan He

China

Sarah Head

UK

Anna Dorothee Heemskerk

Viet Nam

Helen Heffernan

New Zealand

Michael Heggeness

USA

Wiebke Hellenbrand

Germany

Andreas Henke

Germany

Rodrigo T Hernandes

Bouvet Island

Rigoberto Hernandez-Castro

Mexico

Rolando Herrero

France

Helena Hervius Askling

Sweden

Sigrid Heuberger

Austria

Erik Hewlett

USA

Scott Heysell

USA

Matthew Hickman

UK

Geoff Hide

UK

Masaaki Higashiyama

Japan

K High

USA

Sophie Hill

Australia

Andreas Hillenbrand

Germany

Sven Gudmund Hinderaker

Norway

David Hinkle

USA

Yen-Peng Ho

Taiwan

Chi-Lai Ho

Hong Kong

Karin Hoelzer

USA

Ludwig Eduard Hoelzle

Germany

Herbert Hof

Germany

Rodney Hoff

Singapore

Chris Hoffmann

USA

Sven Hoffner

Sweden

Elizabeth Hohmann

USA

Deirdre Hollingsworth

UK

Petter Holme

Sweden

Tollula Honi

South Africa

Russell Hope

UK

Sándor Hornok

Hungary

Maria Hortal

Uruguay

Shervin Hoseini

Iran

Takayuki Hoshina

Japan

Eleanor Hothersall

UK

Ben Howden

Australia

Rebecca Howell-Jones

UK

Peter Hraber

USA

Yu-Hsiang Hsieh

USA

Yongsheng Huang

USA

Jason Huang

Taiwan

Nils-Olaf Hübner

Germany

Mark Hull

Canada

Kristina Hulten

USA

Margit Hummel

Germany

Romney Humphries

USA

Klaus-Peter Hunfeld

Germany

Elizabeth Hunsperger

Puerto Rico

Shirish Huprikar

USA

TakeshiIde

USA

Ramazan Idilman

Turkey

Richard Idro

Uganda

Ikuo Igarashi

Japan

Innocent Ikem

Nigeria

Noboru Imai

Japan

Belen Imperiale

Argentina

Patrik Inderbitzin

USA

Kazuyuki Ishihara

Japan

Tomotada Iwamoto

Japan

Arthur Jackson

USA

Shevin Jacob

USA

Thomas Jacobs

Germany

Fabian Jaimes

Colombia

Seema Jain

USA

Hamid Jalal

UK

Frances Jamieson

Canada

Ruud Jansen

Netherlands

Jean-Paul Janssens

Switzerland

Joseph Jarvis

South Africa

Seyed Mohammad Jazayeri

Iran

Jo Jefferies

UK

Kimberly Jefferson

USA

Claire Jenkins

UK

Neil Jenkins

UK

Adam Jenney

Australia

Jens-Ulrik Jensen

Denmark

Joseph Jeong

South Korea

Peter Jepsen

Denmark

Helene Jeulin

France

Wei Jiang

China

Wolfgang Jilg

Germany

Silvia Jiménez-Jorge

Spain

Fengyi Jin

Australia

Jiyong Jing

China

Mark Jit

UK

Hope Johnson

USA

James Johnson

USA

Atul Johri

India

Adrian Jones

UK

Noyal Joseph

India

Vinod Joshi

India

Ali Judd

UK

Anamarija Jurcev-Savicevic

Croatia

Pontus Jureen

Sweden

Auni Juutilainen

Finland

Rolf Kaiser

Germany

AdrianaKajon

USA

Manish Kakkar

India

Joan Kalyango

Uganda

Andrew Kambugu

Uganda

Santoshi Kamei

Japan

Beate Kampmann

UK

Cheol-In Kang

South Korea

Alan Kaplan

Canada

Beatrix Kapusinszky

USA

Omer Karadag

Turkey

Drosos Karageorgopoulos

Greece

Cigdem Karakukcu

Turkey

Samuel Kariuki

Kenya

John M Karon

USA

Matthew Kasper

USA

Hormuzd Katki

USA

Ghazi Kayali

USA

Karen Keddy

South Africa

Theodoros Kelesidis

USA

Brian Kendall

USA

Dervla Kenna

UK

Gilbert Kersh

USA

Mishal Khan

Pakistan

Lubna Khatoon

Australia

Yury Khudyakov

USA

Muhammad Khurram

Pakistan

Bodin Khwannimit

Thailand

Mogens Kilian

Denmark

Won Yong Kim

South Korea

H. Hyune-Ju Kim

USA

Yae-Jean Kim

South Korea

Bum-Joon Kim

South Korea

Ruth King

UK

Ioannis Kioumis

Greece

Aaron Kipp

USA

Robert Kirkcaldy

USA

Istvan Kiss

UK

Paul Kitsutani

Cambodia

Marc Kiviniemi

USA

Paul Klatser

Netherlands

Terry Klein

USA

Ken Kleinman

USA

Erich Kliewer

Canada

Stephen Knabel

USA

Dave Knox

UK

Nobumichi Kobayashi

Japan

Sándor Kocsubé

Hungary

Uwe Koedel

Germany

Renate Koenig

Germany

Cristiane Koga-Ito

Brazil

Won-Jung Koh

South Korea

Anke Kohlenberg

Germany

Satoshi Koike

Japan

Marin Kollef

USA

Hon Wai Koon

USA

Petros Kopterides

Greece

Izabela Korona-Glowniak

Poland

Titia Kortbeek

Netherlands

Pope Kosalaraksa

Thailand

Andrea Kovacs

USA

Axel Kramer

Germany

Anna Kramvis

South Africa

Lothar Kreienbrock

Germany

Rafal Krenke

Poland

Sushma Krishna

India

Stefan Krueger

Germany

Detlev H Kruger

Germany

Alper Kustarci

Turkey

Ravina Kullar

USA

Nyaradzai EdithKurewa

Zimbabwe

Andrea Kwa

Singapore

Jimmy Kwang

Singapore

Jeff Kwong

Canada

Giovanni La Montagna

Italy

Martin Laass

Germany

Karine Lacombe

France

Shamez Ladhani

UK

F Marc LaForce

USA

Alexander Lai

Hong Kong

Alessia Lai

Italy

Vemu Lakshmi

India

Dora Lam-Himlin

USA

Timothy Landers

USA

Dadja Essoya Landoh

Togo

Christian Lange

Germany

Kamran Lankarani

Iran

Nathanael Lapidus

France

Heidi Larson

UK

Christine Lascols

USA

Cornelia Lass-Floerl

Austria

Irene Latorre

Spain

Eric Lau

Hong Kong

Tsai-Ling Lauderdale

Taiwan

Odile Launay

France

Eduardo Lazcano-Ponce

Mexico

Yann Le Strat

France

Seung Heon Lee

South Korea

Yeongseon Lee

South Korea

Ming-Hsun Lee

Taiwan

Ping-Ing Lee

Taiwan

Vernon Lee

Singapore

Jeong Lee

South Korea

Pyng Lee

Singapore

Alexander Leenders

Netherlands

Christine Lees

USA

Mengistu Legesse

Ethiopia

David Leiby

USA

Qibin Leng

China

Martina Lengerova

Czech Republic

Steven Leonard

USA

Alain Lepape

France

David Leslie

Australia

Toby Leslie

UK

Alan Lesse

USA

Albert Lessing

Australia

Anne-Marie Leuck

USA

Ting Fan Leung

Hong Kong

Elke Leuridan

Belgium

Johannes Levels

Netherlands

Paul Levett

Canada

Anna Levin

Brazil

Myron Levin

USA

Vivian Levy

USA

Rosamund Lewis

Canada

J Lewis

USA

David Lewis

UK

François L'Hériteau

France

Jing Li

USA

Junwen Li

China

Hui Li

China

Xu Li

China

Reto Lienhard

Switzerland

Tore Lier

Norway

Troels Lillebaek

Denmark

Patrick Lillie

UK

Siew Pheng Lim

Singapore

Karla Lima

Brazil

Ming-Wei Lin

Taiwan

Benjamin Linas

USA

Megan Lindley

USA

Mark Lindsley

USA

Marc Lipman

UK

Thiago Lisboa

Spain

Yahong Liu

China

Jinhua Liu

China

Lei Liu

USA

Tao Liu

USA

David Livermore

UK

Unn Ljostad

Norway

Anna Llupià

Spain

Hsiu-Jung Lo

Taiwan

Vincent Lo Re

USA

Mark Lobato

USA

Shawn Lockhart

USA

Sara Lodi

Spain

Li-Cher Loh

Malaysia

Yoon Kong Loke

UK

Guido Lopes dos Santos Santiago

Belgium

Lorena López-Cerero

Spain

Patrizia Lorenzini

Italy

Monica Losi

Italy

Richard Louie

USA

Charito Love

USA

Nicola Low

Switzerland

Frank Lowy

USA

Hongzhou Lu

China

Beibei Lu

USA

Yuanan Lu

USA

Liang Lu

China

Vivian Luchsinger

Chile

Christian Lueck

Germany

Tricia Luhn

USA

Aroonlug Lulitanond

Thailand

Expedito Luna

Brazil

Francoise Lunel Fabiani

France

David Lung

Hong Kong

Fredrick Lutwama

South Africa

Holger Lutz

Germany

David Chien Lye

Singapore

**Edmond M**a

Hong Kong

Frank-Michael Mueller

Germany

Neil Macdonald

UK

CalmanMacLennan

Italy

Jessica MacNeil

USA

Burkhard Madea

Germany

Giordano Madeddu

Italy

Shabir Madhi

South Africa

Venkatesh Madhugiri

India

Marten Maeß

Germany

Namita Mahapatra

India

Ludo Mahieu

Belgium

Rehab Mahmoud Abd El-Baky

Egypt

Hassan Mahomed

South Africa

Anselm Mak

Singapore

Sunny Mak

Canada

Isabelle Malet

France

Josep Mallolas

Spain

Joseph Malone

USA

Helen Maltezou

Greece

Ouattara Mamadou

Cote d'Ivoire

Elias Manavathu

USA

Nicasio Mancini

Italy

Peter Mancuso

USA

Sundhiya Mandalia

UK

Lisa Manhart

USA

Weerawat Manosuthi

Thailand

Pablo ManriqueI-Saide

Mexico

Massoud Mansouri

UK

Lamberto Manzoli

Italy

Fenglou Mao

USA

Camila Marconi

Brazil

Mihai Mares

Romania

Francesca Mariani

Italy

Jose Maria Marimon

Spain

Nikolaos Markou

Greece

Linsey Marr

USA

Fawziah Marra

Canada

Almerico Marruchella

Italy

Jonas Marschall

USA

Kimberly Marsh

UK

Caroline Marshall

Australia

Catherine Marshall

Australia

Loic Martin

France

Anandi Martin

Belgium

Adrian Martineau

UK

Regina Martins

Brazil

Heike Martiny

Germany

Gayane Martirosian

Poland

Joseph Masci

USA

Sundari Mase

USA

Carl Mason

Thailand

Peter Mason

Zimbabwe

Leslie Massad

USA

Robert Massung

USA

Eric Matheson

USA

Catherine Mathews

South Africa

Yasufumi Matsumura

Japan

Alberto Matteelli

Italy

Petri Mattila

Finland

Astrid Mayr

Austria

Teresita Mazzei

Italy

Andrew McBain

UK

James McCaw

Australia

Stephen McGarvey

USA

Rose McGready

Thailand

David McKinsey

USA

Mary-Louise McLaws

Australia

Brian J McMahon

USA

Don McManus

Australia

Ruth McNerney

UK

Anna McNulty

Australia

Takafira Mduluza

Zimbabwe

Luke Mease

USA

Graeme Ayton Meintjes

South Africa

Maria Jose Mellado

Spain

Alexander Mellmann

Germany

Eyal Meltzer

Israel

Maria Cassia Mendes-Correa

Brazil

Godfred Menezes

India

Stefano Merler

Italy

Jessica Merlin

USA

Leonard Mermel

USA

Douglas Scott Merrel

USA

Dominik Mertz

Canada

Muayad Merza

Iraq

Nebiyu Mesfin

Ethiopia

David Mesher

UK

Steven Meshnick

USA

Konradin Metze

Brazil

Agnes Meybeck

France

Nuala Meyer

USA

Elisabeth Meyer

Germany

Sayoki Mfinanga

Tanzania

Argyris Michalopoulos

Greece

Jaap M.Middeldorp

Netherlands

Keren Middelkoop

South Africa

Makoto Miki

Japan

Matthew Mikoleit

USA

Michael Millar

UK

Olivier Mimoz

France

Jesús Mingorance

Spain

Vivi Miriagou

Greece

Saroj Mishra

India

Aruna Mittal

India

Spiros Miyakis

Greece

Naoyuki Miyashita

Japan

Igor Mokrousov

Russia

Kirsten Moller

Denmark

José Moltó

Spain

Susana Monge

Spain

Rosa Monno

Italy

Michael Montalto

Australia

Antonio Montresor

Switzerland

Ginny Moore

UK

Hannah Moore

Australia

Hector Morbidoni

Argentina

Benjamin Mordmueller

Germany

Beatriz Moreira

Brazil

Shunsuke Mori

Japan

Florent Morio

France

Susan Morpeth

Kenya

Giulia Morsica

Italy

Eric Mortensen

USA

Anisa Mosam

South Africa

Chisomo Msefula

Malawi

Philippe Msellati Burkina

Faso

Stephen Mshana

Tanzania

Sia Msuya

Tanzania

Samson Mukaratirwa

South Africa

Asish Mukhopadhyay

India

Fidele Kanyimbu Mukinda

South Africa

Brian Mulhall

Australia

Zuber Mulla

USA

Beat Muller

Switzerland

L. Silvia Munoz-Price

USA

Janna Munster

Netherlands

David Murdoch

New Zealand

Manoj Murhekar

India

RichardMurphy

USA

David Muscatello

Australia

John Muscedere

Canada

Michele Mussap

Italy

Nico Mutters

Germany

Ingomar Mutz

Austria

Monde Muyoyeta

Zambia

Amos Rodger Mwakigonja

Tanzania

Bernard Naafs

Netherlands

Sharon Nachman

USA

Maria Nagel

USA

Jae Hwan Nam

South Korea

Aziz Nather

Singapore

Elena Naumova

USA

Shevanthi Nayagam

UK

Mamadou Ousmane Ndiath

Senegal

Sergey Nejentsev

UK

Martha Nelson

USA

Alexandr Nemec

Czech Republic

Dala Nemenqani

Saudi Arabia

Dionissios Neofytos

USA

Mercy Newman

Ghana

Mun Hon Ng

China

Lai-King Ng

Canada

Hien Nguyen

USA

Audrey Nicholson

UK

Matthias Niedrig

Germany

Eva Moller Nielsen

Denmark

Albert Nienhaus

Germany

Arjen Nikkels

Belgium

Vassilios Nikolaou

Greece

Vladyslav Nikolayevskyy

UK

Ran Nir-Paz

Israel

Hiroshi Nishiura

Hong Kong

John Nkengasong

USA

Carl Erik Nord

Sweden

Abdul Jalil Nordin

Malaysia

Ayman Noreddin

USA

Hans Nothdurft

Germany

SimoneNouer

Brazil

Mary Patricia Nowalk

USA

Francine Ntoumi

Congo

Ulrich Nübel

Germany

Philippe Nwane

Cameroon

Henry Nyamogoba

Kenya

Alan Nyitray

USA

Sheila O'Brien

Canada

Cristina O'Callaghan-Gordo

Spain

Theresa Ochoa

Peru

Abraham Rexford Oduro

Ghana

Shahin Oftadeh

Australia

Dimie Ogoina

Nigeria

Justin O'Grady

UK

Kerry-Ann O'Grady

Australia

Tinuade Ogunlesi

Nigeria

Akira Ohno

Japan

Nobuo Ohta

Japan

ShinichiOka

Japan

Emelda Okiro

Kenya

Isabel Oliver

UK

Sigvard Olofsson

Sweden

Sonja Olsen

USA

Kien Chai Ong

Malaysia

Sylvain Orenga

France

Giovanni Battista Orsi

Italy

Miguel O'Ryan

Chile

Edgar Overton

USA

David Pace

Malta

Maria Clara Padoveze

Brazil

Fabrice Paganin

France

Andrea Page

Canada

Pasquale Pagliano

Italy

Tibor Pál

United Arab Emirates

Domingo Palmero

Argentina

Sarada Panchanathan

USA

Junxiong Pang

Singapore

Efstathia Panotopoulou

Greece

Nitika Pant Pai

Canada

Georgios Pappas

Greece

Roger Paredes

Spain

Shreemanta Parida

India

Subhash Parija

India

Saverio Parisi

Italy

Jorge Parra-Ruiz

Spain

Nicole Parrish

USA

Sonia Passos

Brazil

Gopi Patel

USA

Naimish Patel

India

David Paterson

Australia

Chander Pathak

India

Mical Paul

Israel

Andrew Pavia

USA

Sean Pawlowski

USA

Federico Pea

Italy

Christopher Peacock

Australia

Richard Peek

USA

Frederic Pene

France

Galo Peralta

Spain

Steven Percival

UK

Roman Perez Velasco

Thailand

Olga Perovic

South Africa

Giancarlo Perrone

Italy

Robert Perry

USA

Thomas Peterman

USA

Georg Peters

Germany

Tansy Peters

UK

Eskild Petersen

Denmark

Kyle Petersen

USA

Stephen Peterson

USA

Efi Petinaki

Greece

Georgios Petrikkos

Greece

Linda Petrone

Italy

Melinda Pettigrew

USA

PatrizioPezzotti

Italy

Stephane Picot

France

Kim Picozzi

UK

Christine Pierce Campbell

USA

Antonio Pignatari

Brazil

Carlos Pigrau

Spain

Sandra Pinkert

Germany

Marcello Pinti

Italy

Patrice Piola

Madagascar

Lionel Piroth

France

Rein Jan Piso

Switzerland

Marian Pitts

Australia

Hanna Pituch

Poland

Pedro Plans-Rubio

Spain

Joanne Platell

Australia

Jason Pogue

USA

Carolina Pohl

South Africa

Julien Poissy

France

Eva Polverino

Spain

Yong Poovorawan

Thailand

Corneliu Petru Popescu

Romania

Nurith Porat

Israel

Carolina Porras

USA

Maarten Postma

Netherlands

Garyphallia Poulakou

Greece

Christine Pourcel

France

Spyros Pournaras

Greece

Clydette Powell

USA

Claire Poyart

France

Isobel Mary Poynten

Australia

JoergenPrag

Denmark

Lance Price

USA

Kostas Priftis

Greece

Vincenzo Puro

Italy

Manju Purohit

India

FirdausiQadri

Bangladesh

Flavio Queiroz-Telles

Brazil

Andrea Rachow

Germany

Mohammad Rahbar

Iran

Noordin Rahmah

Malaysia

Alamelu Raja

India

Thandavarayan Ramamurthy

Indonesia

Blandine Rammaert

France

Mary Ramos

USA

Andy Ramsay

Switzerland

Jacqueline Randle

UK

Stephane Ranque

France

Sari Rantala

Finland

Paolo Ravanini

Italy

Giuseppe Ravizzola

Italy

Julian C Rayner

UK

Tim Read

Australia

Terri Rebmann

USA

Gili Regev-Yochay

Israel

Christiane Reichardt

Germany

Frank Reichenberger

Germany

Lisa Reimer

Papua New Guinea

Mark Reinwald

Germany

Richard Reithinger

USA

Jordi Rello

Spain

Franck Remoue

France

Bertrand Renaud

France

Salvador Resino

Spain

Steven Reynolds

USA

Giovanni Rezza

Italy

Mehmood Riaz

Pakistan

Walter Ricciardi

Italy

Guy Richards

South Africa

Jan Hendrik Richardus

Netherlands

Sara Richter

Italy

John Ridderhof

USA

Hans Rieder

Switzerland

Philippe Riegel

France

Leen Rigouts

Belgium

Lee Riley

USA

Felix C. Ringshausen

Germany

Diego Ripamonti

Italy

Viviana Ritacco

Argentina

Caterina Rizzo

Italy

Geert Robaeys

Belgium

Jason Roberts

Australia

Joan Robinson

Canada

Mauricio Rodrigues

Brazil

Jesús Rodríguez-Baño

Spain

Isabel Rodriguez-Barraquer

USA

Eduardo Rodriguez-Noriega

Mexico

Benjamin Rogers

Australia

Emmanuel Roilides

Greece

David Rollinson

UK

Renato Luís Rombaldi

Brazil

Gustavo Romero

Brazil

Lisa Ronald

Canada

John Roord

Netherlands

Warren Rose

USA

Denise Rossato Silva

Brazil

Coleman Rotstein

Canada

Francoise Roudot-Thoraval

France

Francois Rouet

Gabon

Nadine Rouphael

USA

Christian Roussilhon

France

Monika Roy

USA

Qiang Ruan

China

Tobias Rupprecht

Germany

Darren Russell

Australia

William Russell

UK

William Rutala

USA

Daniel Ruzek

Czech Republic

Maria Helena Saad

Brazil

Bradley Sack

USA

Manish Sadarangani

Canada

Anavaj Sakuntabhai

France

Marcel Salathe

USA

Fernando Saldias

Chile

Raquel Sa-Leao

Portugal

Jorge Salluh

Brazil

Marina Salvadori

Canada

Michael Samarkos

Greece

VittorioSambri

Italy

Amidou Samie

South Africa

Sofia Samper

Spain

Orjan Samuelsen

Norway

Jordi Sanchez

Brazil

Johan Sandberg

Sweden

Ingvild Sandøy

Norway

Alessandro Sanduzzi

Italy

Vartul Sangal

UK

Dominique Sanglard

Switzerland

Davison Sangweme

USA

Clemax Sant Anna

Brazil

Sigrid Santos

Brazil

Valeria Saraceni

Brazil

Annalisa Saracino

Italy

Bahador Sarkari

Iran

John Saunders

UK

Esen Savas

Turkey

Anne Savey

France

Robert Sawyer

USA

Hugo Sax

Switzerland

Gianpaolo Scalia Tomba

Italy

Anja Schablon

Germany

Dena Schanzer

Canada

Frieder Schaumburg

Germany

Simone Scheithauer

Germany

Stuart Schembri

UK

Thomas Schiano

USA

Nikolaus Schiering

Switzerland

Maarten Franciscus Schim van der Loeff

Netherlands

Mark Schleiss

USA

Thamarai Schneiders

UK

Paul Schnitzler

Germany

Philipp Schuetz

USA

Mindy Schuster

USA

Eli Schwartz

Israel

Giada Sebastiani

Italy

James Seddon

South Africa

Harald Seifert

Germany

Shamala Devi Sekaran

Malaysia

Elodie Sellier

France

ElenaSeminari

Italy

Arlene Sena

USA

Ian Seppelt

Australia

EmelSesli Cetin

Turkey

LenaSetchanova

Bulgaria

SunilSethi

India

Maninder Singh Setia

India

KwonjuneSeung

USA

Alberto Severini

Canada

**CanSevin**c

Turkey

Leigh Anne Shafer

Canada

Prakeshkumar Shah

Canada

Madhusudana Shampur

India

Isdore CholaShamputa

Canada

Shulian Shang

USA

P Ravi Shankar

Nepal

Fadel A Sharif

Palestinian Territory

Surender K Sharma

India

Kimberly Shea

USA

Crystal Shen

USA

Robert Sherertz

USA

Philip Sherman

Canada

Clive Shiff

USA

Tae Sun Shim

South Korea

Nobuaki Shime

Japan

Gayle Shimokura

USA

Akira Shimouchi

Japan

Lora Shimp

USA

Sonya Shin

USA

Bruce Shiramizu

USA

Norah Shire

USA

Olive Shisana

South Africa

Shmuel Shoham

USA

Andrew Shorr

USA

Farideh Siavoshi

Iran

Antonio Siccardi

Italy

Marco Siccardi

UK

Huma Siddiqui

Norway

Mark Siedner

USA

Enrico M. Silini

Italy

BenjaminSilk

USA

Joao Silva

Brazil

Rosa Maria Silva

Brazil

Arne Simon

Germany

Guus Simons

Netherlands

Gunnar Skov Simonsen

Norway

Steven Singer

USA

Neeloo Singh

India

RosalynSingleton

USA

Nikolaos Sipsas

Greece

Sami Siraj

Pakistan

Sunee Sirivichayakul

Thailand

Seter Siziya

Zambia

Daniel Skiest

USA

Wendy Sligl

Canada

Derek Sloan

Malawi

Marek Smieja

Canada

M. Kumi Smith

USA

Jennifer Smith

UK

Robert William Snow

Kenya

Bolette Soborg

Denmark

Mette Søgaard

Denmark

Kate Soldan

UK

Mark Sonderup

South Africa

Geetika Sood

USA

Lynn Soong

USA

Montse Soriano Gabarro

Germany

Albert Sotto

France

Marc Souris

France

Marcos Sousa

Brazil

Thiago Souza

Brazil

Greg Spear

USA

Roberto Speck

Switzerland

Brad Spellberg

USA

Bryan Spencer

USA

John Spinelli

Canada

Veroniek Spoorenberg

Netherlands

Chandrashekhar Sreeramareddy

Nepal

Padmini Srikantiah

India

Ashok Srinivasan

Uruguay

Sujatha Srinivasan

USA

Gaby Sroczynski

Austria

Marc Steben

Canada

Mariane Stefani

Brazil

Pawel Stefanoff

Poland

MartinSteinau

USA

Florian Steiner

Germany

Edward Stenehjem

USA

Kim Stevens

UK

Ellen Stobberingh

Netherlands

S Stone

UK

Jason Stout

USA

Norval Strachan

UK

Raphael Stricker

USA

Klemen Strle

USA

Birgit Strommenger

Germany

Hugh Sturrock

USA

Juan Suarez

Spain

Nacho Suarez

Spain

Miranda Suchomel

Austria

Sheena Sullivan

Australia

Gerald Sume

Cameroon

Qun Sun

China

Krishna Sundar

USA

Jonas Sunden-Cullberg

Sweden

Yupin Suputtamongkol

Thailand

Michael Surette

Canada

SuheylaSurucuoglu

Turkey

Jayne Sutherland

Gambia

Valentina Svicher

Italy

Sathyamangalam Swaminathan

India

Soumya Swaminathan

India

Frank Tacke

Germany

Muhamed-Kheir Taha

France

Howard Takiff

Venezuela

Kaisar Ali Talukder

Bangladesh

Cheuk Ming Tam

Hong Kong

Paul Tambyah

Singapore

Pranita Tamma

USA

Thuan-Tong Tan

Singapore

Yasuhito Tanaka

Japan

Julian Wei Tze Tang

Singapore

Rosanna Tarricone

Italy

Panayotis T. Tassios

Greece

Norma Tavakoli

USA

Graham Taylor

UK

Jean Tchuenche

USA

René Te Witt

Netherlands

Marc Tebruegge

UK

Paulo Teixeira

Brazil

Laura Temime

France

Edamisan Temiye

Nigeria

Tobias Tenenbaum

Germany

Hwa-Jen Teng

Taiwan

Christian Theilacker

Germany

Nicole Theodoropoulos

USA

Kristof Theys

Belgium

Volker Thiel

Switzerland

Alexander Thielen

Germany

Usa Thisyakorn

Thailand

George Thompson

USA

Kamala Thriemer

Belgium

Franck Thuny

France

Guy Thwaites

UK

Thorsten Thye

Germany

Huai-YuTian

China

Mathuros Tipayamongkholgul

Thailand

Craig Tipple

UK

Lucina Titone

Italy

Kelvin To

Hong Kong

Martin Tolstrup

Denmark

Steven Tong

Australia

Patricia Totten

USA

Aurélie Touron-Bodilis

France

Alberto Tozzi

Italy

Anete Trajman

Brazil

Giorgio Treglia

Italy

Andrea Trevisan

Italy

David Tribble

USA

Chanwit Tribuddharat

Thailand

Srikanth Tripathy

India

Alenka Tropskaza

Slovenia

Iraklis Tsangaris

Greece

Akihito Tsubota

Japan

Paul Tulkens

Belgium

Elaine Tuomanen

USA

Vedat Turhan

Turkey

Nenad Turk

Croatia

Tari Turner

Australia

Jane Turton

UK

Jimmy Twin

Australia

Edet Udo

Kuwait

Stephan Urban

Germany

Yoshihisa Urita

Japan

Pieter Uys

South Africa

Antti Vaheri

Finland

Zol Vaj

Hungary

Violeta Valcheva

Bulgaria

Marta Valenciano

Spain

Francesca Valent

Italy

Louis Valiquette

Canada

Alejandro Vallejo

Spain

Michiel van Boven

Netherlands

Alje Van Dam

Netherlands

David Van de Vijver

Netherlands

Ingrid Viola Francine van den Broek

Netherlands

Pepijn van den Munckhof

Netherlands

Lia van der Hoek

Netherlands

Wim van der Hoek

Netherlands

Els van der Meijden

Netherlands

Marianne A.B. van der Sande

Netherlands

Elske van Gils

Netherlands

Rob van Hest

Netherlands

Jakko van Ingen

Netherlands

Maria Van Kerkhove

UK

Annelies Van Rie

USA

Trevor Van Schooneveld

USA

Susanna Sophiavan Wyk

South Africa

Yvan Vandenplas

Belgium

Wannes Vanderhaeghen

Belgium

Tazio Vanni

UK

Konstantinos Vardakas

Greece

Paula Vaz

Mozambique

Julio Vazquez

Spain

Gonzalo Vazquez-Prokopec

USA

Maria Velasco

Spain

Thirumalaisamy Palanichamy Velavan

Germany

Nienke Veldhuijzen

Netherlands

Inga Velicko

Sweden

Akke Vellinga

Ireland

Henri Verbrugh

Netherlands

Anne Vergison

Belgium

Stefania Vergnano

UK

Rajesh Verma

India

Piero Vernia

Italy

Hans Verstraelen

Belgium

Timo Vesikari

Finland

Dario Vezzani

Argentina

Cecile Viboud

USA

David Vickers

Canada

Jean-Francois Viel

France

Rachel Vieux

France

Gregory Viglianti

USA

Angel Vila-Corcoles

Spain

Paolo Villari

Italy

Gianni Virgili

Italy

VaralakshmiVissa

USA

Heike von Baum

Germany

Andrea von Groll

Brazil

Marie-Louise von Linstow

Denmark

SirendaVong

Cambodia

Andrew Vyse

Belgium

Indra Vythilingam

Malaysia

Atsushi Wada

Japan

Maria Wadl

Germany

John Wain

UK

Mark Wainberg

Canada

Sibongile Walaza

South Africa

Jonas Waldenström

Sweden

Bernhard Walder

Switzerland

Jennifer Walker

Australia

Woolf Walker

UK

Lesley Wallace

UK

Frederic Wallet

France

Jacco Wallinga

Netherlands

DaltonWamalwa

Kenya

Liangxing Wang

China

Shengjun Wang

China

Jiun-Ling Wang

Taiwan

Gui-Qiang Wang

China

Chin-Tien Wang

Taiwan

Jianhua Wang

China

Chen Wang

China

Shih-Min Wang

Taiwan

Hui Wang

China

Ning Wang

China

Jennifer Wang

USA

David Wareham

UK

Roland Weierstall

Germany

Robin Weiss

UK

Maja Weisser

Switzerland

Christian Wejse

Denmark

Cameron Wellard

Australia

Nele Wellinghausen

Germany

Tobias Welte

Germany

Dirk Werber

Germany

Guilherme Werneck

Brazil

Guido Werner

Germany

Marianne Wessling-Resnick

USA

P. Lewis White

UK

Stephen Whitehead

USA

Andrew Christopher Whitelaw

South Africa

Peter Wide

Sweden

Micael Widerstrom

Sweden

Douglas Widman

USA

Nathan Wiederhold

USA

Jeanine Wiener-Kronish

USA

Deborah Williamson

New Zealand

Matthew Willis

USA

Dunja Wilmes

Belgium

Douglas Wilson

South Africa

Peter Wilson

UK

Mark Wilson

USA

Carl Heinz Wirsing von Koenig

Germany

Ferdinand Wit

Netherlands

Jeff Withey

USA

Xavier Wittebole

Belgium

Linda Wittkop

France

Somsri Wiwanitkit

Thailand

Randall Wolcott

USA

Steven Wolf

USA

Michel Wolff

France

Nicole Wolter

South Africa

Sallene Wong

Canada

Karen Wood

USA

Sarah Woodhall

UK

Marta Malgorzata Wroblewska

Poland

Ben-Quan Wu

China

Ming-Shiang Wu

Taiwan

Björn Wullt

Sweden

Bing Xu

China

Lingzhong Xu

China

Kemin Xu

USA

Jianqing Xu

China

Feng Xu

China

Jun Jie Xu

China

TetsuyaYagi

Japan

Javed Yakoob

Pakistan

Teruo Yamashita

Japan

Yang Yang

USA

Hanchun Yang

China

Decheng Yang

Canada

Yonghong Yang

China

Jun Yao

China

Barbara Yawn

USA

Maria Yazdanbakhsh

Netherlands

Sachin Yende

USA

Jung Sook Yeom

South Korea

Solomon A Yime

Norway

Jiehui Kevin Yin

Australia

Dongwan Yoo

USA

Hiroshi Yotsuyanagi

Japan

Fiona Young

UK

Bo Yu

China

Rongbin Yu

China

Hao Yu

USA

Xuejie Yu

USA

Man-Fung Yuen

Hong Kong

Courtney Yuen

USA

Mauro Zaccarelli

Italy

Jean Ralph Zahar

France

Maria Mercedes Zambrano

Colombia

Marta Zancolli

Italy

Hassan Zaraket

USA

Alexandre Zavascki

Brazil

Maurizio Zazzi

Italy

Michele Zeier

South Africa

Jean-Pierre Zellweger

Switzerland

Ming Zeng

China

Yujiang Zhang

China

Wenyan Zhang

China

Shujun Zhang

China

Yanan Zhao

USA

Yadong Zheng

UK

Jilin Zhou

USA

Yi-Hua Zhou

China

Yang Zhou

China

Huachen Zhu

Hong Kong

Marya Zilberberg

USA

Walter Zingg

Switzerland

Thomas Zoller

Germany

Thierry Zozio

Guadeloupe

Aeilko Having Zwinderman

Netherlands

## Pre-publication history

The pre-publication history for this paper can be accessed here:

http://www.biomedcentral.com/1471-2334/13/50/prepub

